# The Effect of Rosuvastatin on Formalin-Induced Nociceptive Behavior in an Experimental Rat Model of Metabolic Syndrome

**DOI:** 10.33549/physiolres.935681

**Published:** 2026-06-01

**Authors:** María Paola Carolina GARCÍA-PAZ, Jorge Skiold LÓPEZ-CANALES, Mireille TOLEDO-BLAS, Antonio FRANCO-VADILLO, Héctor FLORES-HERRERA, Oscar Alberto LÓPEZ-CANALES, María Cristina PAREDES-CARBAJAL, Jair LOZANO-CUENCA

**Affiliations:** 1 Servicio de Pediatría, Hospital General Regional No. 25, Instituto Mexicano del Seguro Social, Ciudad de México, México; 2 Departamento de Fisiología y Desarrollo Celular, Instituto Nacional de Perinatología, Ciudad de México, México; 3 Sección de Estudios de Posgrado e Investigación, Escuela Superior de Medicina de Instituto Politécnico Nacional, Ciudad de México, México; 4 Departamento de Inmuno-Bioquímica, Instituto Nacional de Perinatología, Ciudad de México, México; 5 Departamento de Fisiología. Facultad de Medicina, Universidad Nacional Autónoma de México, Ciudad de México, México

**Keywords:** High-fat diet, Nociception, Metabolic syndrome, Formalin test

## Abstract

The aim of this study was to determine the effect of rosuvastatin (10 mg/kg/day, p.o.) on the flinching nociceptive behavior produced by subcutaneous injection of 0.5 % formalin in the dorsum of the right hind paw from Wistar rats submitted to a 16-week treatment, including a standard rat chow diet (STD-diet) or a high fat-diet (HF-diet). At three days post-treatment, the formalin-induced nociceptive response was assessed for 1 h. Compared to the STD-diet, the HF-diet significantly increased the number of flinches in the second phase of the formalin test, as well as inducing higher body weight and levels of glucose, total cholesterol, triglycerides, insulin and blood pressure. Rosuvastatin significantly decreased the formalin-induced nociceptive behavior after both diets and significantly reduced those metabolic parameters in rats with a HF-but not STD-diet.

## Introduction

Metabolic syndrome is a confluence of several disorders, which can include hyperglycemia, compensatory hyperinsulinemia, insulin resistance, dyslipidemia and a systemic proinflammatory state [1]. Numerous studies suggest that hyperglycemia, hyperinsulinemia, insulin resistance and dyslipidemia are each importantly related to the control of pain [2–3]. There are reports on the involvement of hyperglycemia in the development of tactile allodynia and hyperalgesia in experimental models of diabetes in rats [4], hyperinsulinemia in the production of hyperalgesia and autophagy in rats [5], insulin resistance in the development of peripheral neuropathy in humans [6] and dyslipidemia in the development of neuropathy in mice [2].

Whereas the aforementioned disorders are known to participate in susceptibility to pain, few studies have focused on the role of metabolic syndrome as a whole in this sense. According to recent clinical and experimental evidence, metabolic syndrome could contribute to an increase in the prevalence of polyneuropathy and the intensity of chronic pain [7–10]. However, its contribution to the degree of acute inflammatory pain remains unclear. Hence, the present study set out to investigate the effect of a High-fat diet (HF-diet) and/or the daily administration of rosuvastatin on nociceptive behavior. First, we compared the effects of a STD- and HF-diet, the latter of which is an experimental model of metabolic syndrome [11], on the nociceptive behavior produced by the injection of formalin into the dorsal surface of the right hind paw of adult male Wistar rats. Then we explored the effect of the chronic administration of rosuvastatin, a 3-hydroxy-3-methyl glutaryl-coenzyme A reductase inhibitor with lipid-lowering properties [12] on the nociceptive behavior of animals fed either a STD- or HF-diet.

## Material and Methods

### Animals

Experiments were performed on adult (4-week-old) male Wistar rats (100–110 g). The animals (n=48), obtained from our breeding facilities, had free access to food and drinking water during the 16-week treatment. They were housed in plastic cages in a special temperature-controlled room (22±2 °C, 50 % humidity) on a 12:12 h light/dark cycle (lights on at 7 am). The protocol was approved by the Animal Care Committee of our institution (Instituto Nacional de Perinatología, Mexico City, Mexico), and is in accordance with the U.K. Animals (Scientific Procedures) Act, 1986 and associated guidelines of the British Parliament: http://www.legislation.gov.uk/ukpga/1986/14/contents (accessed on May 15, 2025).

### The model of metabolic syndrome

Animals were randomly distributed into two groups given: (i) a SD-diet containing 51 % carbohydrate, 4 % fat and 21 % protein (n=24) or (ii) a HF-diet with 33 % ground commercial rat chow, 33 % full fat sweetened condensed milk (Nestlé), 7 % sucrose and 27 % water (n=24), as previously reported [11]. These diets lasted 16 weeks.

### The measurement of nociceptive activity

Three days after the 16-week treatment ended, antinociception was assessed by using the formalin test [13]. First, the rats were placed in open acrylic observation chambers and allowed 30 min to acclimate to their surroundings. They were then removed for formalin administration. Fifty microliters of diluted formalin (0.5 %) were injected s.c. into the dorsal surface of the right hind paw with a 30-gauge needle. The animals were returned to the chambers and immediately the responses to nociceptive pain were quantified, a process facilitated by the placement of mirrors. Accordingly, the number of flinches of the injected paw was counted during 1-min periods, which were recorded every 5 min for 1 h post-injection [14]. Flinching was readily discriminated, characterized as a rapid and brief withdrawal or a flexing of the injected paw. Formalin-induced flinching behavior was biphasic [13–14]. Between the initial acute phase (0–10 min) and a prolonged persistent response (15–60 min), there was a relatively short quiescent period. At the end of the experiment the rats were sacrificed in a CO_2_ chamber.

### Drugs

Rosuvastatin (a gift from AstraZeneca, Mexico City) was dissolved in distilled water. Fresh solutions were prepared for each experiment.

### Study design

Protocol 1: The effect of a HF-diet on nociceptive behavior was investigated in two groups of rats (n=8 per group). The animals were submitted to a 16-week STD- or HF-diet. Three days after the respective diet was over, nociceptive behavior was evaluated by the formalin test.

Protocol 2: The effect of rosuvastatin and a HF-diet on nociceptive behavior was examined in four groups of rats (n=8 per group). Each group was submitted to a 16-week treatment, including a STD- or HF-diet plus a daily oral dose of rosuvastatin (10 mg/kg/day, p.o.) or the vehicle (water; 2 ml/kg/day, p.o.). Accordingly, the groups were as follows: 1) a STD-diet plus vehicle, 2) a STD-diet plus rosuvastatin, 3) a HF-diet plus vehicle, and 4) a HF-diet plus rosuvastatin. The following metabolic parameters were determined the day after the treatments ended: body weight, glucose, total cholesterol, triglycerides, insulin and blood pressure. Nociceptive behavior was assessed by the formalin test.

### Data analysis and statistics

Data (from 8 animals in each group) are expressed as the mean ± SEM. Curves were constructed by plotting the number of flinches as a function of time. The area under the curve (AUC), an expression of the duration and intensity of the effect, was calculated by the trapezoidal rule. An increase in the number of flinches or the AUC is herein described only for the second phase of the formalin test, since no significant effect was detected in the first phase. Differences between the metabolic and nociceptive parameters of the four groups in protocol 2 were examined with two-way analysis of variance (ANOVA) followed by the Holm-Sidak *post hoc* test. Differences between the nociceptive parameters of the two groups in protocol 1 were examined by one-way ANOVA. Statistical difference was considered at P<0.05.

## Results

### Nociceptive responses in rats with a HF-diet

**Fig. 1:**
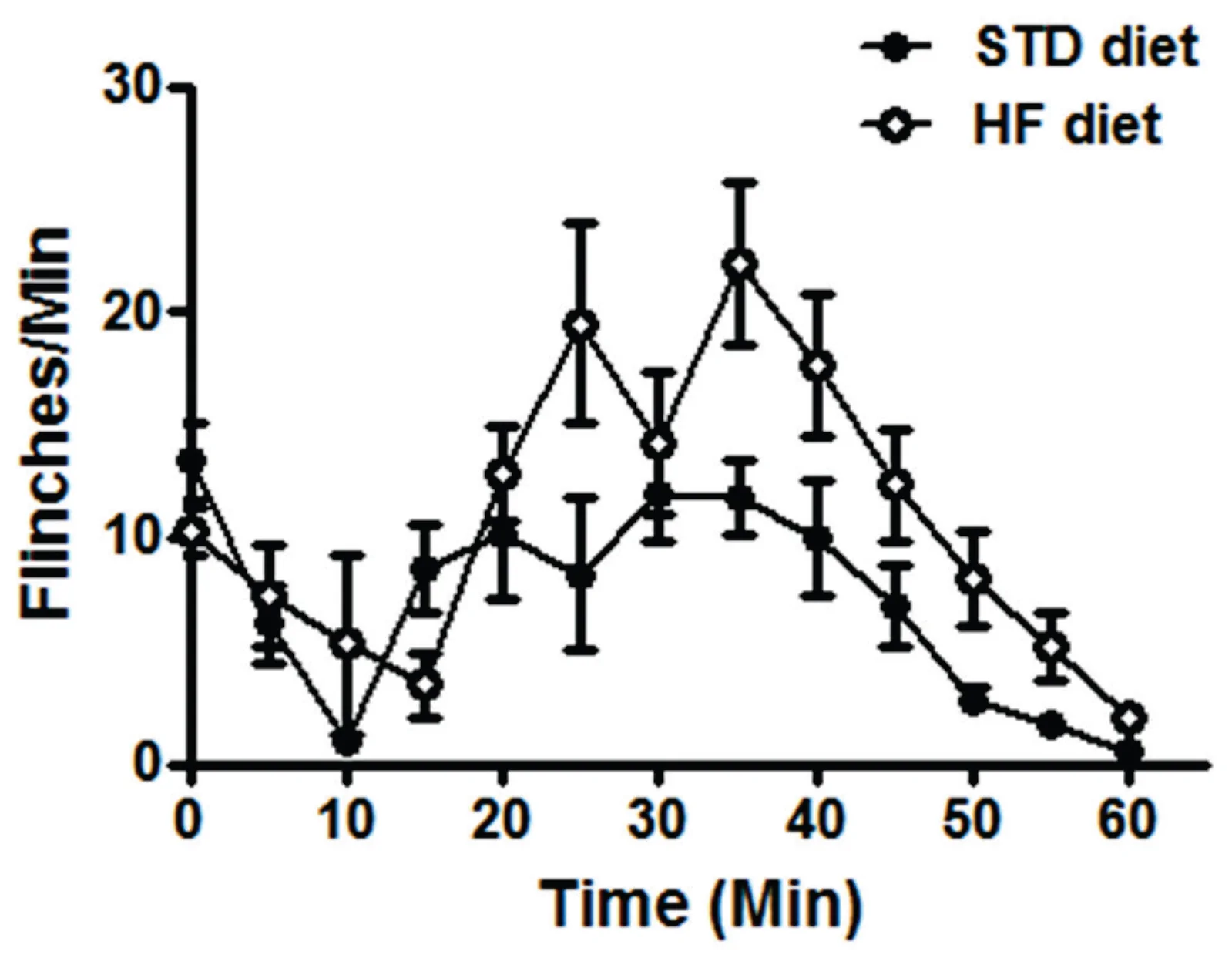
Time course of the nociceptive effect produced by a subcutaneous injection of 0.5 % formalin in the dorsum of the right hind paw of rats given a 16-week standard (STD)-diet (solid circles) or high fat (HF)-diet (hollow circles). Data from 8 animals per group are presented as the mean ± SEM.

The subcutaneous injection of 0.5 % formalin in the dorsum of the right hind paw of rats with a STD-diet resulted in a typical biphasic pattern of flinching behavior (Fig. 1; solid circles), as previously reported [13]. Phase 1 of the nociceptive response began immediately after the formalin injection and then declined gradually during approximately 10 min. Phase 2 began about 15 min post-injection and lasted until the end of the observation period. Compared to the STD-diet, the HF-diet caused more frequent formalin-induced flinching in the second phase (595.83±56.71* vs. 166.00±34.62; * P<0.05), but not in the first phase (43.25±11.04 vs. 76.67±15.90; Fig. 1, hollow circles). Only in the second phase did the difference between the total number of flinches in the two groups reach the level of significance.

### Effect of a HF-diet plus rosuvastatin on the metabolic parameters

**Fig. 2:**
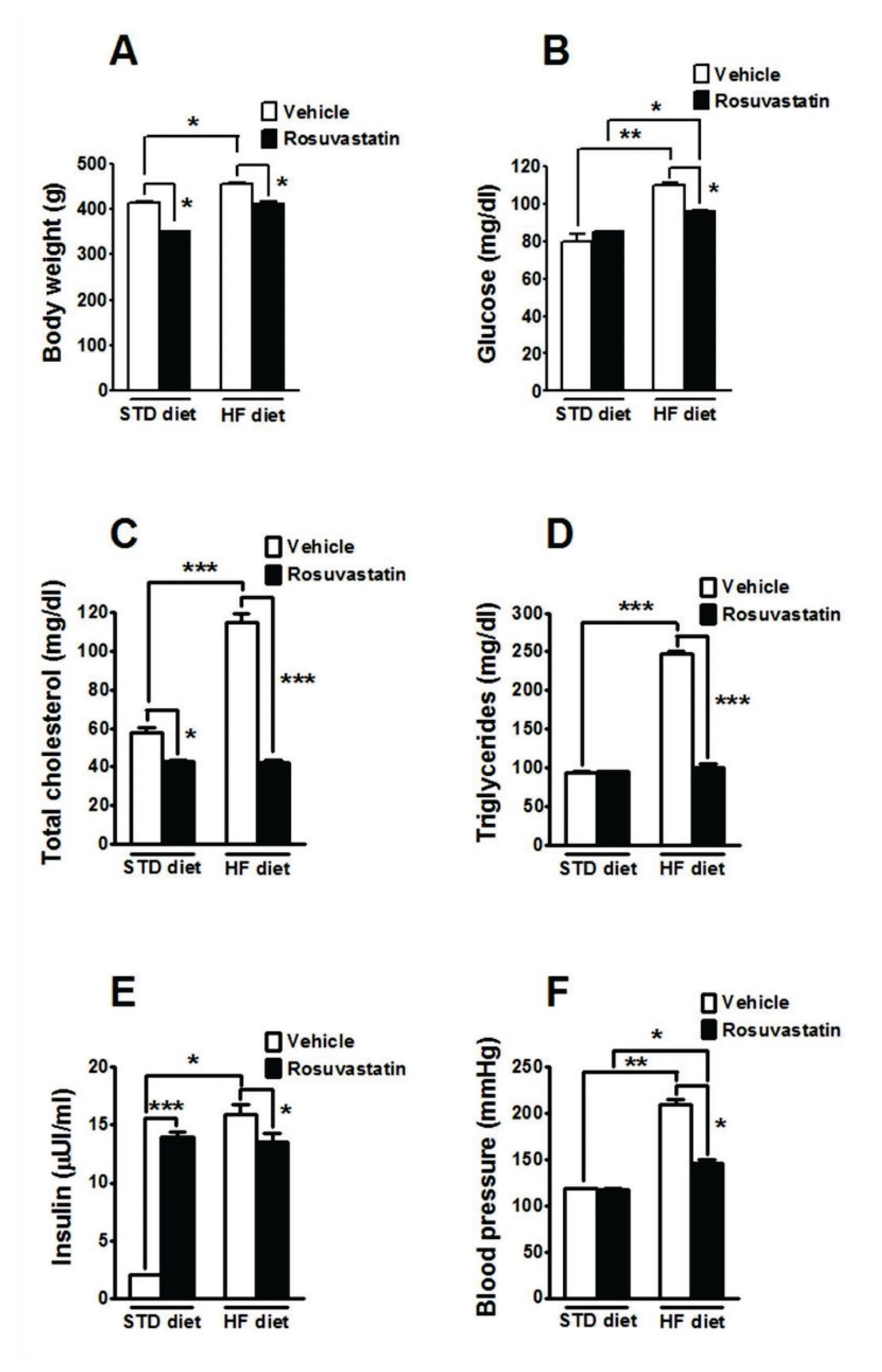
Body weight (**A**), glucose (**B**), total cholesterol (**C**), triglycerides (**D**), insulin (**E**) and blood pressure (**F**) of male Wistar rats following a 16-week treatment consisting of a standard (STD)- or high fat (HF)-diet plus the administration of the vehicle or rosuvastatin. Data from 8 animals per group are expressed as the mean ± SEM and were analyzed with two-way ANOVA followed by the Holm-Sidak *post hoc* test. * P<0.05, ** P<0.01, *** P<0.001.

Rats were fed a STD- or HF-diet and treated daily with the vehicle (2 ml/kg/day, p.o.) or rosuvastatin (10 mg/kg/day, p.o.) during 16 weeks. Concerning the vehicle-treated groups, the HF-diet (vs. STD) significantly (P<0.05) increased all metabolic parameters in rats. Regarding the HF-diet, the treatment with rosuvastatin (vs. the vehicle) significantly (P<0.05) decreased all metabolic parameters (Fig. 2).

### Effect of the vehicle or rosuvastatin on nociceptive behavior in rats with a STD- or HF-diet

**Fig. 3:**
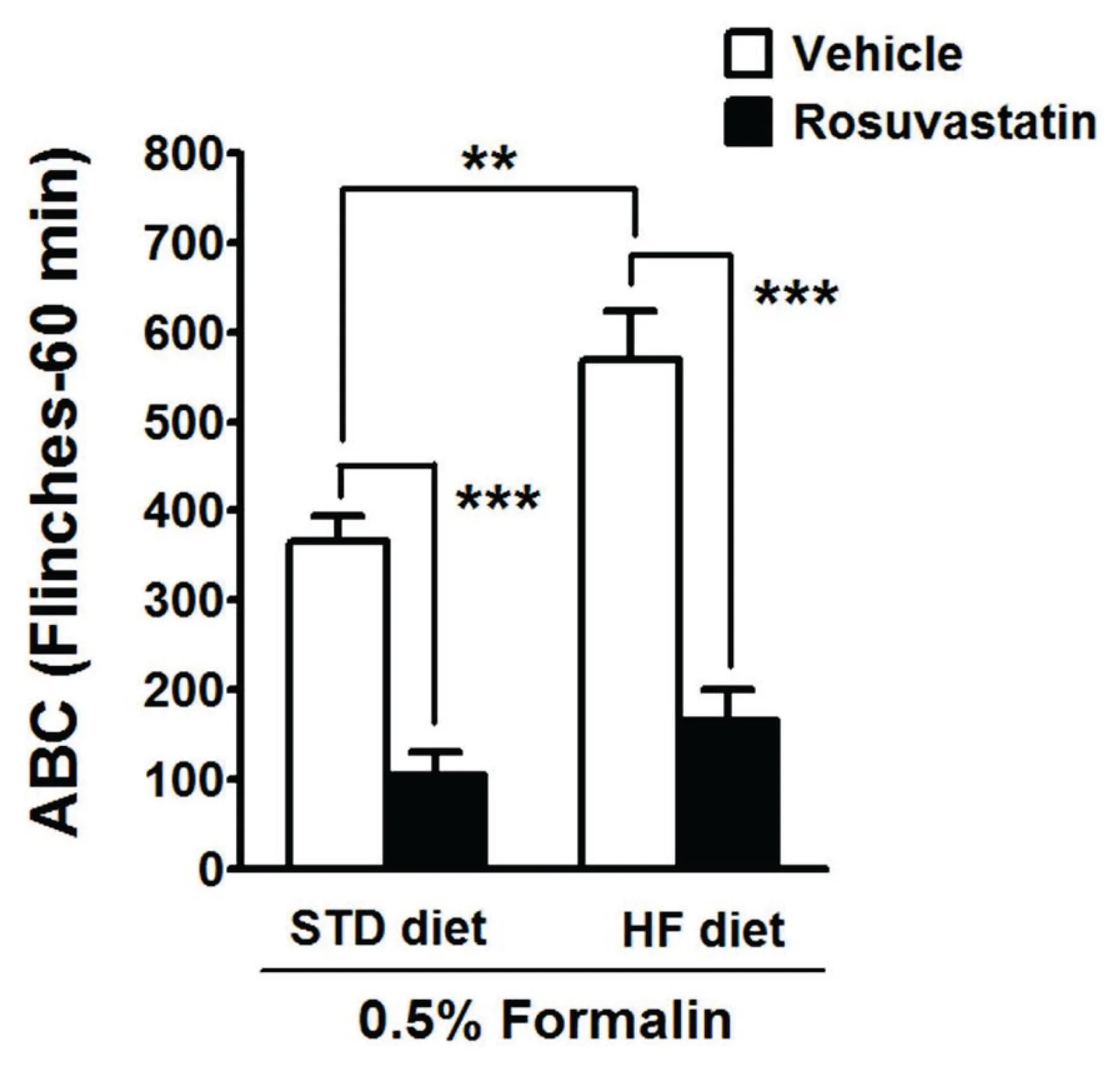
Comparison between the effect of the vehicle (2 ml/kg/day) and rosuvastatin (10 mg/kg/day) on the number of flinches produced by the subcutaneous injection of 0.5 % formalin in rats. The 16-week treatment included oral administration of the vehicle or rosuvastatin and a standard (STD)- or high fat (HF)-diet. Data from 8 animals per group are expressed as the mean ± SEM and were analyzed by two-way ANOVA followed by the Holm-Sidak *post hoc* test. ** P<0.01, *** P<0.001.

Rats were submitted to a 16-week treatment, including a daily dose of the vehicle (water; 2 ml/kg/day, p.o.) or rosuvastatin (10 mg/kg/day, p.o.) concomitant with a STD- or HF-diet. Subsequently, a nociceptive response was provoked (Fig. 3). According to the two-way ANOVA analysis of nociceptive behavior: (i) rats fed a HF-diet (vs. standard) had a significantly greater nociceptive response in the second phase of the formalin test (P<0.01); (ii) the administration of rosuvastatin (vs. the vehicle) produced a significant antinociceptive effect (P<0.001) in rats fed either diet; (iii) a significant difference (P<0.01) existed between the vehicle-treated rats fed the STD and HF-diets; (iv) there was no significant difference (P=0.223) for rosuvastatin-treated animals between the STD and HF-diet groups; and (v) no significant interaction was shown between diet and treatment (P=0.085).

## Discussion

### Nociceptive responses in rats with a STD-diet vs. HF-diet

A 16-week HF-diet was capable of inducing obesity in rats and significantly higher levels of glucose, total cholesterol, triglycerides, insulin and blood pressure (compared to the animals with a STD-diet), as previously reported [11]. Additionally, this diet significantly augmented the flinching behavior of animals in the formalin test.

The results from this experimental model of pain are consistent with recent experimental and clinical findings. Accordingly, it has been found that: (i) a HF-diet from the time of weaning until 20 weeks of age facilitates the development of trigeminal sensitization to capsaicin in mice [15]; (ii) a 17-week HF-diet increases pain-like behaviors produced by an intra-articular injection of Complete Freund’s Adjuvant in mice [8]; (iii) metabolic syndrome can be directly related to the intensity of knee pain in patients with abdominal obesity, independently of age, sex and weight [9]; and (iv) metabolic syndrome heightens the prevalence of polyneuropathy and impairs peripheral nerve function [7]. Thus, it has been widely reported that metabolic syndrome plays an important role in upregulating the degree and prevalence of chronic pain. The present data suggest that metabolic syndrome may intensify acute inflammatory pain.

We previously performed two pilot studies of pain in rats fed a HF-diet during 8 and 12 weeks. In both cases, the HF-diet (vs. standard) fostered significantly higher body weight gain and levels of glucose, total cholesterol, triglycerides and insulin [11]. However, the HF-diet for 8 or 12 weeks did not modify flinching behavior (data not shown). Hence, 8 weeks of an HF-diet proved to be sufficient to significantly elevate the metabolic parameters, but 12 weeks of this diet was insufficient to trigger more frequent paw-flinching responses to pain. In the present study, a 16-week HF-diet was able to heighten the nociceptive response, exacerbating hyperalgesia and flinching behavior elicited by the formalin injection.

Although there are no well-established mechanisms that could explain the greater frequency of nociceptive behavior caused by a HF-diet, there are some interesting insights in literature. A 12-week HF-diet leads to greater carrageenan-induced thermal hyperalgesia in rats, involved in the downregulation of activity of spinal peroxisome proliferator-activated receptors (PPARs)α and an elevated expression of inflammatory mediators and nuclear factor-κB [16]. In mice fed a HF-diet (but not STD), a facial injection with a low dose of capsaicin, a TRPV1 agonist, produces a rise in c-FOS immuno-reactivity, a neuronal activation marker, in the trigeminal nucleus caudalis [15]. A 6-month HF-diet fosters higher systemic concentrations of pro-inflammatory cytokines such as IL-6 and TNF-α in C57BL/6J mice [17].

### Effect of a HF-diet and rosuvastatin on metabolic parameters

In rats treated with the vehicle, a 16-week HF-diet (vs. STD) significantly increased body weight (Fig. 2A) and levels of glucose (Fig. 2B), total cholesterol (Fig. 2C), triglycerides (Fig. 2D), insulin (Fig. 2E) and blood pressure (Fig. 2F). This result reinforces previous reports of an 8-week HF-diet augmenting the same metabolic parameters in male Wistar rats [11] and of an HF-diet being linked to greater concentrations of glucose, cholesterol, triglycerides and insulin [8,18].

In relation to rosuvastatin, on the other hand, the use of this drug and other statins to treat dyslipidemia is well known. Among the rats presently fed the HF-diet, those given the rosuvastatin treatment (vs. the vehicle) exhibited significantly lower body weight (Fig. 2A), glucose (Fig. 2B), total cholesterol (Fig. 2C), triglycerides (Fig. 2D) and blood pressure (Fig. 2F). In accordance with the latter finding is the proposal that statins be considered candidate drugs for treating complex metabolic disorders [18]. The rosuvastatin-induced attenuation of weight gain observed herein (Fig. 2A) coincides with a recent report suggesting that a 6-week rosuvastatin treatment tended to diminish mean weight gain in mice fed a HF-diet. In that study, rosuvastatin treatment decreased the weight of fatty tissue samples and led to significantly less fat storage in adipose tissue samples [18].

For rats with a standard diet, rosuvastatin also resulted in lower body weight compared to vehicle-treated animals (Fig. 2A). The ability of rosuvastatin to reduce body weight, which is not dependent on a HF-diet, may be related to a previously described decline in plasmatic levels of leptin prompted by pravastatin [19]. This result was detected in a model of spontaneously developing type II diabetes mellitus (DM II) using Otsuka Long-Evans Tokushima Fatty rats. Pravastatin significantly decreased the progressive elevation of plasma leptin, and significantly lowered body weight and food intake [19]. Certainly, we have no clear-cut explanation about the mechanisms underlying the reduced body weight fomented by rosuvastatin administration. The inclusion here of the aforementioned reports is speculative and can only serve to provide some orientation for future research. Additional experiments, beyond the scope of this study, are needed to clarify the possible involvement of leptin in the ability of rosuvastatin to reduce body weight.

The glucose-lowering effect of rosuvastatin (compared to the effect of the vehicle) in rats fed a HF-diet (Fig. 2B) coincides with the decreased fasting glucose levels in mice generated by rosuvastatin treatment (3 mg/kg/day, for 45 consecutive days) concomitant with diet-induced obesity [18]. However, the effect of statins on the control of glycemia and the incidence of diabetes mellitus is controversial. On one hand, the subchronic treatment of mice with atorvastatin during diet-induced obesity (10 mg/kg/day, for 45 consecutive days) improves fasting glucose levels [18], and pravastatin (100 mg/kg/day) retards the progression of glucose intolerance in a model of spontaneously developed DM II [19]. On the other hand, a few clinical studies show that rosuvastatin (20 mg/day) and atorvastatin (80 mg/day) increase the risk of new-onset diabetes mellitus [20–21], which is apparently linked to the use of a relatively high dose [22]. Concerning the rats herein fed a standard diet, the chronic treatment with rosuvastatin did not modify glucose levels (vs. the vehicle). Hence, the activity of this lipid lowering drug (at the dose currently administered) is probably related to glucose homeostasis in the present experimental model.

The cholesterol-lowering effect of rosuvastatin in rats fed either a STD or HF-diet (Fig. 2C) can be attributed to its well-known capacity to inhibit 3-hydroxy-3-methyl glutaryl coenzyme A reductase. The latter enzyme is involved in the novo synthesis of cholesterol in liver, ultimately enhancing the expression of LDL receptors, which in turn increase cholesterol uptake from the blood [12]. Likewise, the ability of rosuvastatin to reduce triglycerides (Fig. 2D) is in accordance with previous clinical studies. For instance, 5, 10 and 20 mg rosuvastatin taken once daily diminished triglycerides by 30.1 %, 30.1 % and 32.3 %, respectively [23]. Additionally, 24 weeks of treatment with 10 mg rosuvastatin caused a highly significant decrease in serum triglycerides of patients with metabolic syndrome [24].

In rats fed a STD-diet, the drastic rise in insulin levels produced by rosuvastatin (vs. the vehicle) (Fig. 2E) coincides with previous reports suggesting that some statins can impair insulin secretion and/or exacerbate insulin resistance [25–27]. On the other hand, when comparing the two groups receiving the vehicle, rats with the HF-diet displayed a high diet-induced level of insulin. Moreover, when comparing the two groups of animals fed a HF-diet, the rosuvastatin treatment prompted a moderate but significant insulin-lowering effect (Fig. 2E). The latter is consistent with previous studies, in which, C5BL/6J female- and C57BL/6J male-mice fed a HF-diet developed insulin resistance and increased insulin output. In both groups of animals, rosuvastatin treatment reduced compensatory insulin release [28–29]. Salunkhe *et al*. [28], suggest that rosuvastatin improved the insulin sensitivity, but impaired the Ca^2+^ signaling in beta cells that regulates insulin release. Yao *et al*. [29] suggest that rosuvastatin may improve insulin sensitivity by altering the gut microbiota and encouraging the synthesis of butyric acid.

For rats fed an HF-diet, the lowering of blood pressure resulting from the rosuvastatin (vs. vehicle) treatment (Fig. 2F) is consistent with the significant statin-induced reduction in the diastolic blood pressure found in adult patients [30], as well as with the cardiovascular pleiotropic effects attributed to statins [31–33]. The mechanisms of these effects are unknown and require further investigation.

### Effect of rosuvastatin on the hyperalgesia produced by a HF-diet

The antinociceptive effect of the daily oral administration of rosuvastatin in rats fed a STD-diet (Fig. 3) is in agreement with previous studies in which rosuvastatin: (i) significantly inhibited the second but not the first phase of formalin-induced nociception [33]; (ii) significantly diminished the number of writhes prompted by an intraperitoneal injection of acetic acid in mice [33]; (iii) prevented the development of mechanical and thermal sensitivity generated in partial sciatic nerve-ligated mice [34], and (iv) prevented the mechanical sensitivity of the lower lip in rats suffering from mental nerve chronic constriction injury [34]. However, the antinociceptive effect of rosuvastatin on formalin-induced nociception was assessed by Ghaisas *et al*. [33] one hour after its acute oral administration. In our experiments, contrarily, the response of rats to formalin was evaluated three days after finishing the rosuvastatin treatment. This protocol excluded the immediate antinociceptive effects resulting from the acute administration of the drug, measuring only the effect produced by its chronic administration.

Regarding the biphasic nature of formalin-induced nociception, the first phase entails direct stimulation of sensory nerve fibers, thus representing neuropathic pain. Contrarily, the underlying mechanism of pain during the second phase is inflammation mediated by prostaglandins, histamine, serotonin, bradykinin and cytokines such as IL-1β, IL-6, TNF-α, eicosanoids and NO [35–37]. Hence, the fact that rosuvastatin inhibited the second but not the first phase of formalin-induced paw-flinching behavior suggests the participation of inflammatory mediators. In partial support of this idea, treatment with rosuvastatin reduced the local inflammation produced by the injection of formaldehyde into a subcutaneous region in rats [33]. Formaldehyde-induced paw edema is mediated by an early release of substance P and bradykinin (neurogenic phase), followed by a tissue mediated response involving the release of histamine, serotonin, prostaglandins and bradykinin [38]. Moreover, rosuvastatin exhibits significant antinociceptive and anti-inflammatory activities in acute and chronic models of pain and inflammation, respectively [33]. Rosuvastatin abolished IL-1β expression in peripheral nerves, which had been triggered by ligation. [34] A pharmacological blockade of IL-1β can prevent or attenuate neuropathic pain [39].

On the other hand, it has been proposed that rats with or without obesity exhibit increased pain behaviors when on the HF-diet. Direct effects on the nervous system could be part of the pain-inducing mechanism of a HF-diet. An HF-diet increases macrophage infiltration of the dorsal root ganglion (DRG) [40], as found in a local DRG inflammation model [41] and a study of chemotherapy-related pain [42]. The importance of direct effects in nerve tissue is also supported by recent reports suggesting that diet-induced obesity generates downregulation of spinal PPARα. This would facilitate susceptibility to a peripheral inflammatory challenge by enhancing the inflammatory response in the spinal cord, thus intensifying peripheral inflammation and inflammatory hyperalgesia in obesity [43].

Although studies have described the antinociceptive and anti-inflammatory effects of rosuvastatin in acute and chronic models of pain and inflammation, the original contribution of the current study is the finding that rosuvastatin alleviates the hyperalgesia stemming from a HF-diet. This is the first report, to our knowledge, of a significantly greater frequency of flinching behavior (by animals in the formalin test) caused by a 16-week HF-diet (Fig. 3), and of a significant decrease in the HF-diet-induced hyperalgesia produced by the daily oral administration of rosuvastatin during the same treatment period.

Concerning the attenuation by rosuvastatin of the hyperalgesia promoted by the HF-diet, the underlying mechanisms are unclear. However, there are some insights into the possible mechanisms in literature. Thus, hyperglycemia induced in a diabetic rat model produced a reduction in foot withdrawal latency in a standard thermal stimulus model, treatment with rosuvastatin 20 mg/kg corrected this reduction in foot withdrawal latency [44]. In this sense, it has been proposed that statins can attenuate thermal hyperalgesia in diabetic mice by inhibiting the production of mevalonate and isoprenoids, which in turn inhibits the translocation of RhoA/ROCK (the former is a small GTPase protein, and the latter a Rho-associated protein kinase) [45].

Besides, PPARs might be essential for both nociceptive transmission- and antinociceptive effects of statins-in metabolic syndrome. As was already noted, there is evidence that suggests diet-induced obesity causes spinal PPARα to be downregulated, which exacerbates peripheral inflammation and inflammatory hyperalgesia [43]. Moreover, some studies suggest that stimulation of PPARγ can modulate nociceptive transmission in inflammatory and neuropathic pain [46–47]. It has been suggested that there are molecular cross-talk pathways between statins and PPARs that could be involved in their anti-inflammatory properties. Statins activate PPAR through ERK1/2 and p38 MAPK-dependent COX-2 production in macrophages [48]. Also in macrophages, a cross-talk between PPAR and statin is seen. In these cells, the acute anti-inflammatory effects of statins involve PPAR-*via* inhibition of the PKC signaling pathway [49].

Finally, indirect evidence exists for the possible attenuation of reactive oxygen species as part of the neuropathic ameliorative actions of statins. Numerous studies have demonstrated the critical role of oxidative stress derived from NADPH oxidase 2 (NOX2) in the initiation and maintenance of neuropathic pain [50–51]. Furthermore, treatment with different statins has been shown to reduce the level of reactive oxygen species in endothelial cells [52]. Significant statin-mediated increases in the enzymatic activity of thioredoxin (through S-nitrosylation) may be responsible for a significant decrease in intracellular reactive oxygen species [50].

It is important to note that the aforementioned is speculative, and that additional research into the mechanisms underlying the antinociceptive effects of statins in metabolic syndrome is outside the purview of this study.

## Conclusions

A 16-week HF-diet caused a significantly greater frequency of paw-flinching nociceptive behavior in the second phase of the formalin test, as well as elevated levels of the following metabolic parameters: body weight, glucose, total cholesterol, triglycerides, insulin and blood pressure. These parameters were all significantly diminished when adding the daily oral administration of rosuvastatin (10 mg/kg) to the 16-week HF-diet. Thus, the use of rosuvastatin offers a therapeutic alternative for inhibiting the development of hyperalgesia in patients with metabolic syndrome.
